# Novel Pituitary Actions of Epidermal Growth Factor: Receptor Specificity and Signal Transduction for *UTS1*, *EGR1*, and *MMP13* Regulation by EGF

**DOI:** 10.3390/ijms20205172

**Published:** 2019-10-18

**Authors:** Qiongyao Hu, Shaohua Xu, Cheng Ye, Jingyi Jia, Lingling Zhou, Guangfu Hu

**Affiliations:** Hubei Provincial Engineering Laboratory for Pond Aquaculture, College of Fisheries, Huazhong Agricultural University, Wuhan 430070, China; HQY960819@163.com (Q.H.); xsh2018308@163.com (S.X.); yechenging@163.com (C.Y.); Jiajy94@163.com (J.J.); 15527699872@163.com (L.Z.)

**Keywords:** signal transduction, pituitary cells, pharmacological test, ErbB, grass carp

## Abstract

Epidermal growth factor (EGF) is a member of the EGF-like ligands family, which plays a vital role in cell proliferation, differentiation, and folliculogenesis through binding with EGF receptors, including ErbB1 (EGFR/HER1), ErbB2 (HER2), ErbB3 (HER3), and ErbB4 (HER4). In mammals, many functional roles of EGF have been reported in the ovaries and breasts. However, little is known about the functions of EGF in the pituitary, especially in teleost. In this study, using grass carp pituitary cells as the model, we try to examine the direct pituitary actions of EGF in teleost. Firstly, transcriptomic analysis showed that 599 different expressed genes (DEGs) between the control and EGF-treatment group were mainly involved in cell proliferation, cell migration, signal transduction, and transcriptional regulation. Then, we further confirmed that EGF could significantly induce *UTS1*, *EGR1*, and *MMP13* mRNA expression in a time-and dose-dependent manner. The stimulatory actions of EGF on *UTS1* and *EGR1* mRNA expression were mediated by the MEK_1/2_/ERK_1/2_ and PI_3_K/AKT/mTOR pathways coupled with both ErbB1 and ErbB2 in grass carp pituitary cells. The receptor specificity and signal transductions for the corresponding responses on *MMP13* mRNA expression were also similar, except that the ErbB2 and PI_3_K/AKT/mTOR pathway were not involved. As we know, *MMP13* could release EGF from HB-EGF. Interestingly, our data also showed that the MMPs inhibitor BB94 could suppress EGF-induced *UTS1* and *EGR1* mRNA expression. These results, taken together, suggest that the stimulatory actions of EGF on *UTS1* and *EGR1* mRNA expression could be enhanced by EGF-induced *MMP13* expression in the pituitary.

## 1. Introduction

Epidermal growth factor (EGF) is a small protein of 6 kDa containing 53 amino acids, which comprises three disulfide bridges [1]. The biological effects of EGF are mediated mainly through four tyrosine kinase receptors, namely ErbB1 (HER1), ErbB2 (HER2), ErbB3 (HER3), and ErbB4 (HER4) [2]. EGF is a potent mitogen growth factor, and so it is involved in the process of cell growth, differentiation, proliferation, metabolism, and tumorigenesis [3]. The EGF ligand and receptor could also play an important role in the renewal of stem cells in early embryonic development, skin, liver, and gut [4]. In the hypothalamus–pituitary–adrenal (HPA) axis, EGF could regulate adrenocorticotropic hormone (ACTH) release through the up-regulation of hypothalamic corticotropin releasing hormone (CRH) [5]. At the pituitary level, EGF could stimulate luteinizing hormone (LH) release [6] and gonadotrope mitosis [7] in rats, and even increase plasma follicle-stimulating hormone (FSH) and LH levels in vivo in ewes [8]. In addition, EGF could also induce prolactin (PRL) synthesis and reduce growth hormone (GH) synthesis in rat pituitary tumor cells [9].

In zebrafish, previous studies found that EGF could significantly enhance the final maturation of the oocytes [10]. Further studies found that EGF was predominantly expressed in the oocytes, whereas epidermal growth factor receptor (EGFR) was highly detected in the follicle cells, which suggested that EGF was a potential paracrine/juxtacrine factor from the oocytes to regulate the function of the follicle cells [11]. At the pituitary level, previous studies found that the EGFR could be detected in zebrafish pituitary cells, but EGF had no effect on the expression of FSHβ, LHβ, and GH [12]. Recently, our study found that EGF could significantly induce somatolactin α (SL α) and tachykinin receptor 3 (TACR3) secretion and synthesis in grass carp pituitary cells [13]. Besides, little is known about the direct pituitary actions of EGF in teleost.

To further examine the direct pituitary actions of EGF in teleost, the primary cultured grass carp pituitary cells were used as the model. Firstly, the global pituitary actions of EGF were examined by using the RNA-Seq technique. Secondly, we further investigated the receptor specificity and signal pathways for EGF-induced Urotensin1 (*UTS1*) and early growth response 1 (*EGR1*) mRNA expression in grass carp pituitary cells. Thirdly, we also examined the direct pituitary actions of EGF in matrix metallopeptidase 13 (*MMP13*) and tissue inhibitor of metalloproteinase 3 (*TIMP3*) gene expression. Finally, we further examined the functional role of *MMP13* in EGF-induced *UTS1* and *EGR1* gene expression in pituitary cells.

## 2. Results

### 2.1. Transcriptomic Analysis

**Figure 1:**
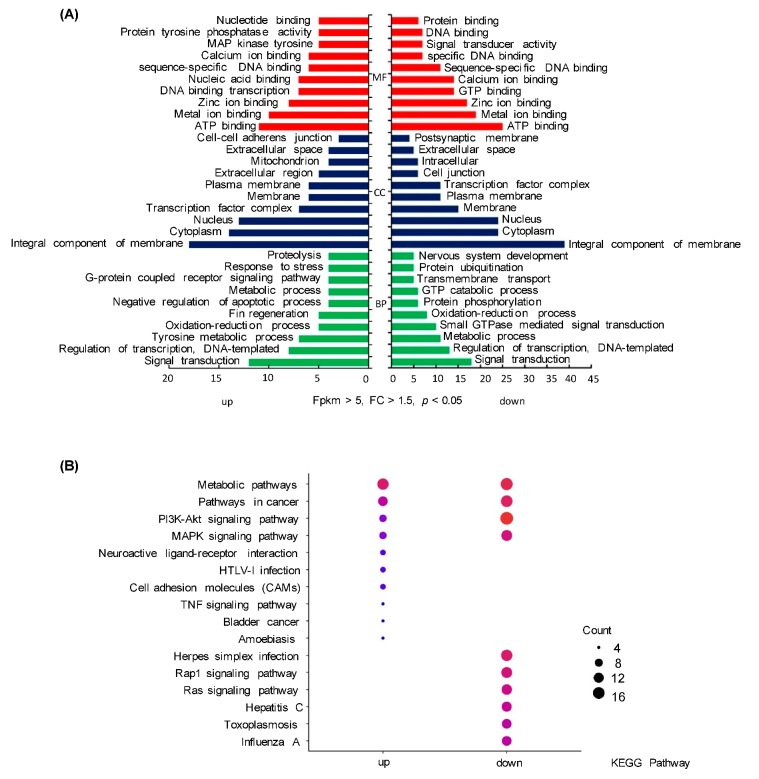
Gene ontology (GO) and Kyoto Encyclopedia of Genes and Genomes (KEGG) analysis. (**A**) GO classification of the assembled differential expression genes (DEGs) of grass carp pituitary cells into molecular function, biological function, cellular component. (**B**) KEGG pathway enrichment analysis for DEGs in grass carp pituitary. Statistics of the top 10 enriched pathways for DEGs of up and down regulation. Up, up-regulated genes; down, down-regulated genes; count, the number of DEGs.

**Figure 2:**
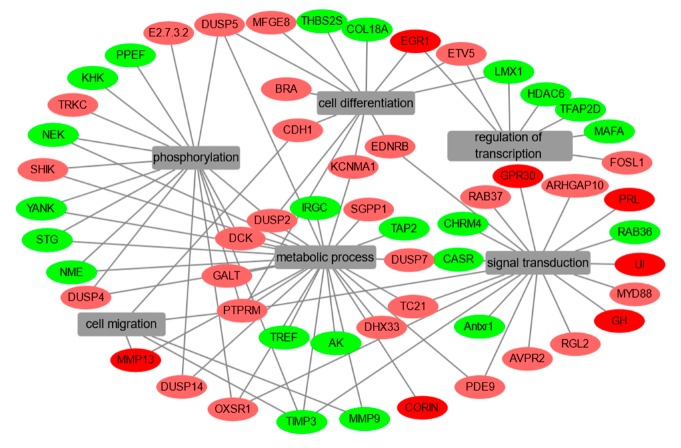
DEGs were enriched in the biological process of cell migration, cell differentiation, signal transduction, metabolic process, phosphorylation, and regulation of transcription in grass carp pituitary cells cultured by EGF treatment. Red indicates that the gene is increased, green indicates the gene is decreased in abundance relative to the control group and grey in the caption indicates the categories of biological process.

To investigate the global regulation of EGF in fish pituitary, a high-throughput transcriptome was used to compare the transcript levels between the control and EGF-treatment groups. In total, 19,486 genes were identified in grass carp pituitary cells. Compared to the control group, 599 differential expression genes (DEGs) were detected after EGF (0.5 μM)-treatment, fragments per kilobase of exon per million fragments mapped (FPKM) > 5, *p* < 0.05, fold change (FC) > 1.5, including 195 up-regulated DEGs and 404 down-regulated genes. GO analysis showed that these DEGs were classified in three main ontologies, including cellular component, biological process, and molecular function (Figure 1A). Within the cellular component category, the ‘integral component of membrane’, ‘cytoplasm’, ‘nucleus’, ‘transcription factor complex’, ‘membrane’, and ‘plasma membrane’ were the most enriched GO terms (Figure 1A). In addition, the most abundant groups in molecular function were ‘ATP binding’, ‘metal ion binding’, ‘zinc ion binding’, ‘GTP binding’, and ‘DNA binding transcription’ (Figure 1A). Finally, the GO enrichment analysis of biological process revealed that the top 46 up-regulated DEGs (Table 1) and top 48 down-regulated DEGs (Table 2) were involved in cell migration, cell differentiation, signal transduction, metabolic process, phosphorylation, and transcriptional regulation (Figure 2).

To further understand the direct pituitary functions of EGF, annotated pathways of DEGs were analyzed using the Kyoto Encyclopedia of Genes and Genomes (KEGG) database. The results revealed that a total of 209 DEGs were enriched in the top 10 pathways. Among them, the up-regulated DEGs were mostly enriched in ‘metabolic pathways’ and ‘pathways in cancer’, and the down-regulated DEGs were mainly enriched in ‘PI_3_K–Akt signaling pathway’, ‘metabolic pathway’, and ‘pathways in cancer’ (Figure 1B).

### 2.2. EGF-Induced Critical DEGs in Grass Carp Pituitary Cells

**Figure 3:**
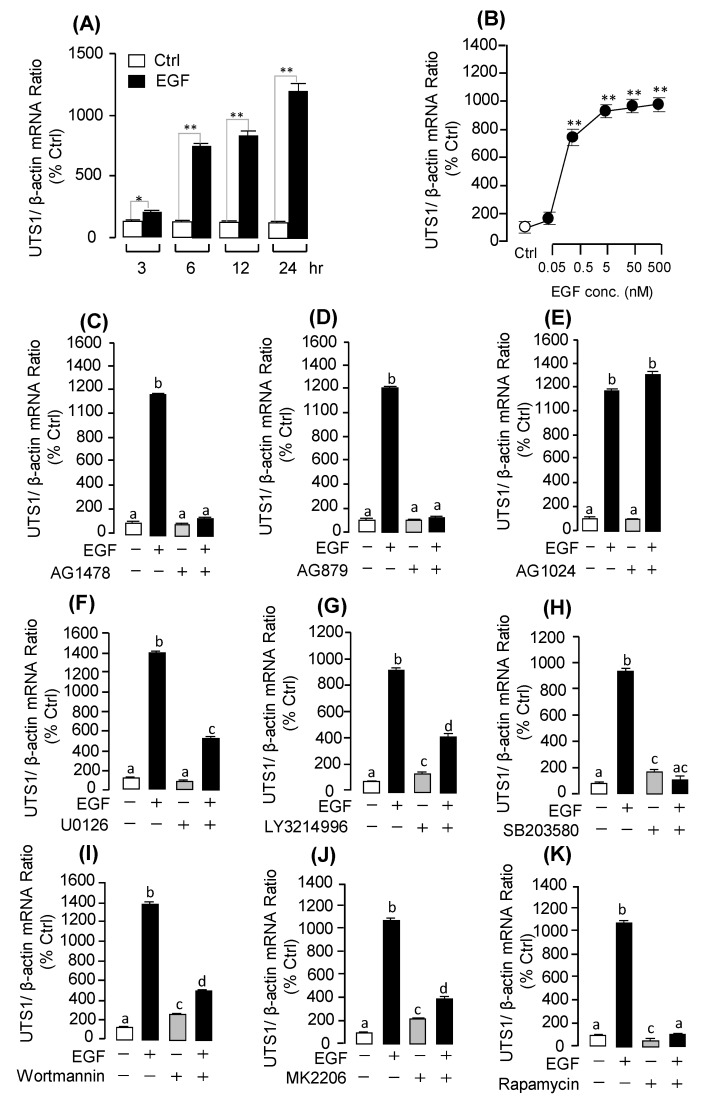
Synergistic effects of EGF on *UTS1* mRNA expression and receptor specificity and post-receptor signal pathway of EGF (0.5 μM)-induced *UTS1* mRNA expression in grass carp pituitary cells. (**A**) Time course of EGF (0.5 μM) treatment on *UTS1* mRNA expression. (**B**) Effect of EGF concentration (0.05–500 nM)-induced on *UTS1* mRNA expression in grass carp pituitary cells. (**C**–**E**) Effects of ErbB1 antagonist AG1478, ErbB2 antagonist AG879, and IGF receptor antagonist AG1024 on EGF-induced *UTS1* mRNA expression, respectively. (**F**–**H**) The effects of EGF (0.5 μM) induced *UTS1* mRNA transcription with the MEK inhibitor U0126 (10 μM), ERK1/2 inhibitor LY3214996 (10 μM), and p38MAPK inhibitor SB203580, respectively. (**I**–**K**) Co-treatment with the PI_3_K inhibitor Wortmannin (10 μM), AKT inhibitor MK2206 (10 μM), and mTOR inhibitor Rapamycin (10 μM) on EGF (0.5 μm)-induced *UTS1* mRNA expression for 24 h, respectively. After drug treatment, total RNA was isolated and used for real-time PCR of *UTS1* mRNA expression. The differences between groups were considered as significant at *p* < 0.05 (“*”) or highly significant at *p* < 0.01 (“**”). The groups denoted by different letters represent a significant difference at *p* < 0.05.

**Figure 4:**
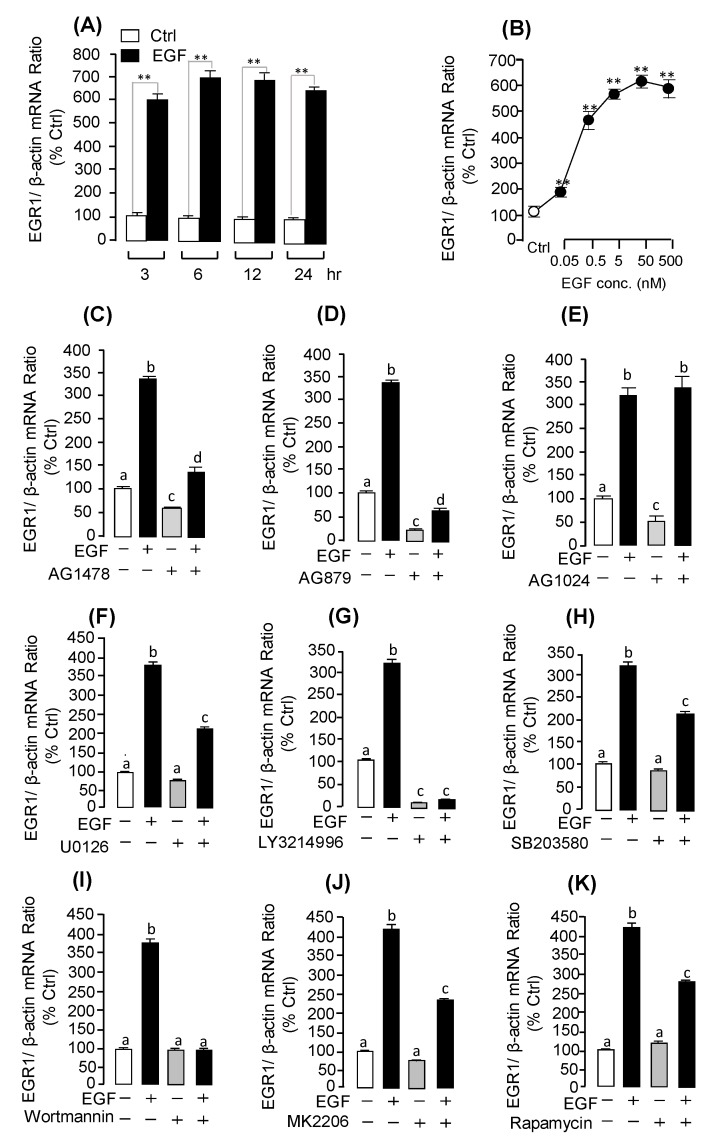
EGF induced *EGR1* mRNA expression in grass carp pituitary cells, including receptor specificity and signal transduction pathways. (**A**) In the time course experiment, pituitary cells were treated with EGF (0.5 μM). (**B**) In the dose experiment, pituitary cells were cultured with EGF (0.05–500 nM). (**C**–**E**) Receptor specificity of EGF (0.5 µM)-induced *EGR1* mRNA expression; effects of ErbB1 antagonist AG1478 (10 µM), ErbB2 antagonist AG879 (10 µM), and IGF receptor antagonist AG1024 (10 µM) on *EGR1* mRNA expression for 24 h, respectively. (**F**–**H**) Signal transduction of *EGR1* mRNA expression induced by EGF (0.5 μM) in grass carp pituitary cells. The effects of *UTS1* mRNA transcription induced by EGF (0.5 μM) with EGF (0.5 μM) in the presence or absence of the MEK inhibitor U0126 (10 μM), ERK1/2 inhibitor LY3214996 (10 μM), or p38MAPK inhibitor SB203580 (10 μM), respectively. (**I**–**K**) The effects of EGF (0.5 μM) induced *EGR1* mRNA expression with the PI_3_K inhibitor Wortmannin (10 μM), AKT inhibitor MK2206 (10 μM), or mTOR inhibitor Rapamycin (10 μM) by EGF (0.5 μM)-induced *EGR1* mRNA expression for 24 h, respectively. After drug treatment, total RNA was isolated and used for real-time PCR of *UTS1* mRNA expression. The differences between groups were considered as highly significant at *p* < 0.01 (“**”). The groups denoted by different letters represent a significant difference at *p* < 0.05.

**Figure 5:**
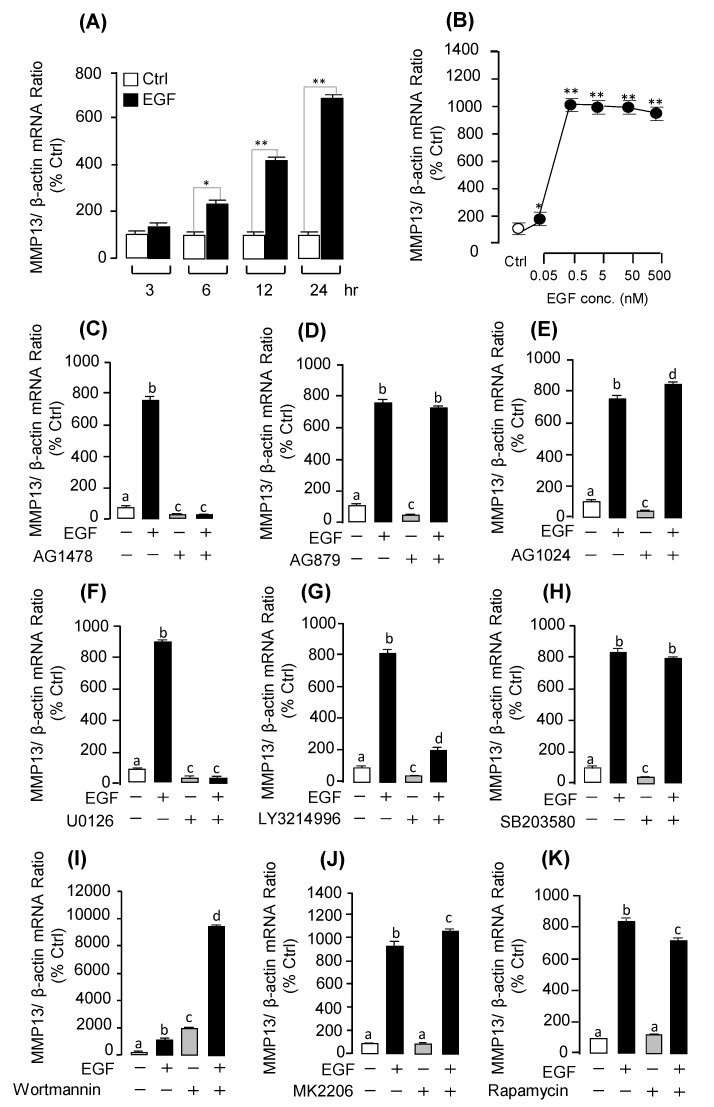
EGF induced *MMP13* mRNA expression and receptor specificity and signal transduction mechanism in grass carp pituitary cells. (**A**) Pituitary cells were treated with EGF (0.5 μM) in a time dependent manner. (**B**) dose-dependent manner of EGF (0.05–500 nM) induced *MMP13* mRNA expression, respectively. (**C**–**E**) Effects of ErbB1 antagonist AG1478 (10 µM), ErbB2 antagonist AG879 (10 µM), and IGF receptor antagonist AG1024 (10 µM) on *MMP13* mRNA expression for 24 h, respectively. (**F**–**H**) Signal transduction of EGF-induced *MMP13* mRNA expression in grass carp pituitary cells. Co-treatment of 24 h with the MEK blocker U0126 (10 μM), ERK1/2 inhibitor LY3214996 (10 μM), or p38MAPK inhibitor SB203580(10 μM) induced *MMP13* mRNA expression was examined in grass carp pituitary cells, respectively. (**I**–**K**) Co-treatment of 24 h with the PI_3_K inhibitor Wortmannin (10 μM), AKT inhibitor MK2206 (10 μM), and mTOR inhibitor Rapamycin (10 μM) induced *MMP13* mRNA expression was examined, respectively. After drug treatment, total RNA was isolated for real-time PCR of *MMP13* mRNA expression. The differences between groups were considered as significant at *p* < 0.05 (“*”) or highly significant at *p* < 0.01 (“**”). The groups denoted by different letters represent a significant difference at *p* < 0.05.

Among the DEGs identified by RNA-Seq analysis, we focused on three up-regulated DEGs: *MMP13*, *EGR1*, and *UTS1*. To further confirm these DEGs, we incubated the grass carp pituitary cells with EGF to detect their mRNA expression by real-time PCR. The results showed that EGF could significantly induce pituitary *UTS1* (Figure 3A; Supplementary Figure S1A), *EGR1* (Figure 4A; Supplementary Figure S1B), and *MMP13* (Figure 5A; Supplementary Figure S1C) mRNA expression in a time-course dependent manner. In the dose-dependent experiment, the results showed that the transcript levels of *UTS1* (Figure 3B), *EGR1* (Figure 4B), and *MMP13* (Figure 5B) were steadily increased with increasing concentrations of EGF (0.5–500 nM). 

### 2.3. Receptor Specificity and Signal Transduction for EGF-Induced UTS1 and EGR1 Gene Expression

In this experiment, a pharmacological approach was recruited to clarify the receptor specificity for EGF-induced *UTS1* and *EGR1* gene expression. The results showed that the up-regulation of *UTS1* and *EGR1* mRNA expression was consistently observed in grass carp pituitary cells with EGF treatment for 24 h. These stimulatory effects on *UTS1* mRNA expression could be totally abolished by co-treatment with the ErbB1 antagonist AG1478 (Figure 3C; Supplementary Figure S2A) or ErbB2 antagonist AG879 (Figure 3D; Supplementary Figure S2E), while the Insulin-like growth factor I receptor (IGF-IR) antagonist AG1024 was not effective in this regard (Figure 3E). Similarly, the stimulatory effects of EGF on *EGR1* mRNA expression could be also blocked by simultaneous incubation with ErbB1 antagonist AG1478 (Figure 3C; Supplementary Figure S2B) or ErbB2 antagonist AG879 (Figure 3D; Supplementary Figure S2F) but not the IGF-IR antagonist AG1024 (Figure 3E).

To further elucidate the post-receptor signaling mechanism involved in the up-regulation of *UTS1* and *EGR1* mRNA expression by EGF, various pharmacological inhibitors targeting different signaling pathways were recruited. As a first step, cotreatment with the MEK1/2 inhibitor U0126, ERK1/2 inhibitor LY3214996, or p38MAPK inhibitor SB203580 could all block the stimulatory effects of EGF on *UTS1* (Figure 3F,G,H; Supplementary Figure S3A) and *EGR1* (Figure 4F,G,H; Supplementary Figure S3C) mRNA expression. In the parallel experiments, EGF-induced *UTS1* and *EGR1* expression were also tested with the inhibitors for individual components of the PI_3_K/AKT/mTOR pathway. In this case, EGF-induced *UTS1* (Figure 3I,J,K; Supplementary Figure S3B) and *EGR1* (Figure 4I,J,K; Supplementary Figure S3D) mRNA expression could be suppressed/totally abolished by co-treatment with the PI_3_K inhibitor Wortmannin, AKT inhibitor MK2206, or mTOR inhibitor Rapamycin. 

### 2.4. Receptor Specificity and Signal Transduction for EGF-Induced MMP13 mRNA Expression

To clarify the receptor specificity and signal transduction for EGF-induced *MMP13* mRNA expression, a pharmacological approach was used. As shown in Figure 5, the stimulatory effects of EGF on *MMP13* could be blocked by simultaneous incubation with ErbB1 antagonist AG1478 (Figure 5C; Supplementary Figure S2C), but not ErbB2 antagonist AG879 (Figure 5D; Supplementary Figure S2G) and the IGF-IR antagonist AG1024 (Figure 5E). With the use of pharmacological blockers targeting different signaling pathways, the signal transduction mechanisms for the up-regulation of *MMP13* mRNA expression were examined. The stimulatory effects of EGF on *MMP13* mRNA expression were notably dispelled by simultaneous incubation with the MEK1/2 inhibitor U0126 (Figure 5F; Supplementary Figure S3E), or ERK inhibitor LY3214996 (Figure 5G), but not with the PI_3_K inhibitor Wortmannin (Figure 5I; Supplementary Figure S3F), AKT inhibitor MK2206 (Figure 5J), or mTOR inhibitor Rapamycin (Figure 5K). To further confirm whether MEK/ERK cascades were involved in EGF-induced post-receptor signaling, the effects of EGF and EGFR inhibitor AG1478 treatment on ERK phosphorylation were tested in grass carp pituitary cells. As shown in Supplemental Figure S4, EGF could significantly induce the phosphorylation of ERK in grass carp pituitary cells. In addition, EGFR inhibitor AG1478 could significantly block EGF-induced ERK phosphorylation. 

### 2.5. Functional Role of MMP13 in EGF-Induced UTS1 and EGR1

**Figure 6:**
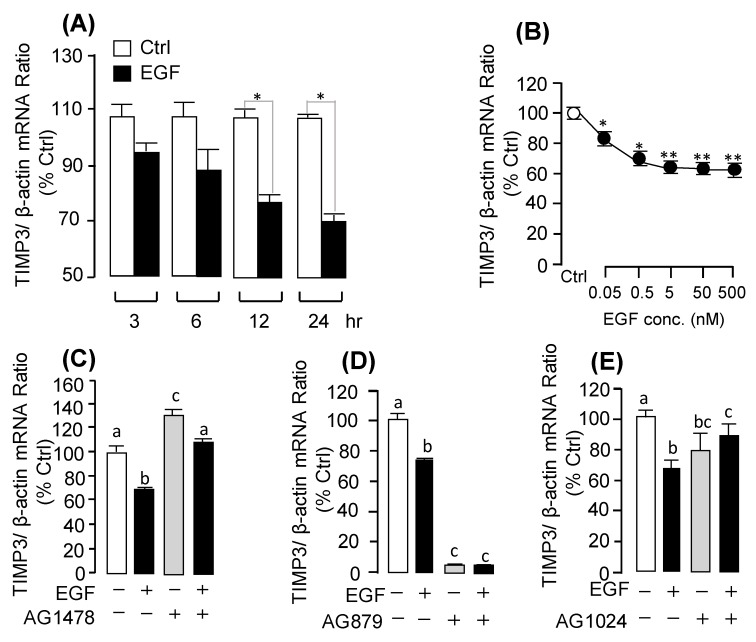
EGF induced *TIMP3* mRNA expression and receptor specificity in grass carp pituitary. (**A**) Time course of EGF (0.5 μM) treatment on *TIMP3* mRNA expression. (**B**) Effect of EGF concentration (0.05–500 nM)-induced on *TIMP3* mRNA expression in grass carp pituitary cells. (**C**–**E**) Effects of ErbB1 antagonist AG1478 (10 µM), ErbB2 antagonist AG879 (10 µM), and IGF receptor antagonist AG1024 (10 µM) on *TIMP3* mRNA expression for 24 h, respectively. After drug treatment, total RNA was isolated for real-time PCR of *MMP13* mRNA expression. In the data present (mean ± SEM), the differences between groups were considered as significant at *p* < 0.05 (“*”) or highly significant at *p* < 0.01 (“**”). The groups denoted by different letters represent a significant difference at *p* < 0.05.

**Figure 7:**
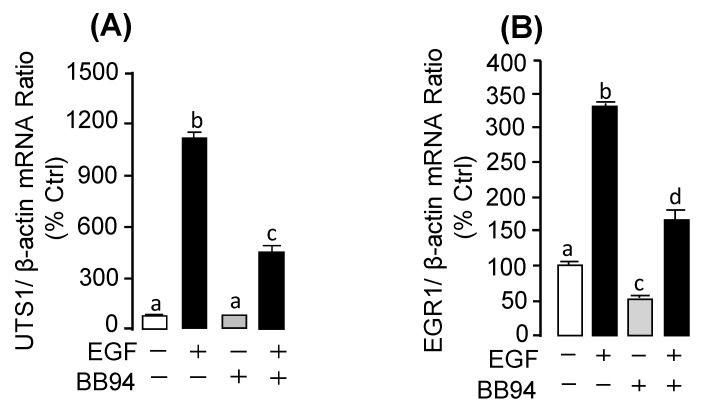
The functional role of in EGF-induced *UTS1* and *EGR1* in grass carp pituitary. (**A**) Effect of the inhibitor of MMPs BB94 (10 µM) on *UTS1* mRNA expression. (**B**) Effect of the inhibitor of MMPs BB94 (10 µM) on *EGR1* mRNA expression. After drug treatment, total RNA was isolated for real-time PCR of *UTS1* and *EGR1* mRNA expression. In the data present (mean ± SEM), the differences between groups were considered as significant at *p* < 0.05 with different letters.

Previous studies have reported that *TIMP3* is the endogenous inhibitor for *MMP13*. Interestingly, our present study found that EGF could inhibit the pituitary *TIMP3* mRNA expression in a time-course (Figure 6A; Supplementary Figure S1D) and dose-dependent manner (Figure 6B). For the receptor specificity, the EGF-inhibited *TIMP3* mRNA expression could be recovered by co-treatment with ErbB1 antagonist AG1478 (Figure 6C), but not with the ErbB2 antagonist AG879 (Figure 6D) or IGF-IR antagonist AG1024 (Figure 6E). Furthermore, the EGF-induced *UTS1* or *EGR1* mRNA expression could be abolished by co-treatment with MMP inhibitor BB94 (Figure 7A,B). 

## 3. Discussion

Previous studies have reported that EGF could play an important role in mammalian pituitary [14]. However, little is known about the pituitary actions of EGF in lower vertebrate. Grass carp (*Ctenopharyngodon idellus*) is the most important aquaculture species in China, with a total production of 5.50 million tonnes in 2018 [15]. As we know, the pituitary is the crucial organ for the regulation of reproduction and growth in teleost, so it will be important to clear the pituitary actions of EGF in teleost. Using grass carp as a model, transcriptomic analysis showed that EGF could induce 195 genes and inhibit 404 genes. These DEGs were involved in cell migration, cell differentiation, signal transduction, metabolic process, phosphorylation, and transcriptional regulation. Similarly, in mammals, previous studies have also reported that EGF could regulate several pituitary functions, including cell proliferation, cell migration [7], and gland tumorigenesis [16].

CRH plays an important role in the HPA system in regulating stress physiology [17]. *UTS1* was firstly isolated and purified from white sucker and common carp [18]. Further studies found that fish *UTS1* had a closed structural and biological homology with ovine CRH and the frog skin peptide sauvagine [19]. In addition, the *UTS1* could also induce ACTH release in mammalian and fish pituitary [20]. Previous studies have found that EGF could induce hypothalamic CRH release [5]. In the present study, we found that EGF could directly induce *UTS1* mRNA expression in grass carp pituitary cells. These results suggest that EGF could also be involved in stress physiology mediated by *UTS1* in grass carp pituitary. In addition, our present study also found that EGF could induce *EGR1* mRNA expression in grass carp pituitary cells. *EGR1* is a member of the immediate early gene family of transcription factors, which could regulate a wide variety of transcripts [21]. *EGR1* could also be stimulated by many environmental signals, including growth factors [22]. A previous study reported that EGF-induced *EGR1* expression could be involved in the down-regulation of matrix metallopeptidase 9 (MMP9) expression in lymphoma cells [23]. These results suggest that EGF could induce *EGR1* expression to regulate several physiological functions in teleost pituitary.

*MMP13*, also called collagenase 3, is a member of the matrix metalloproteinase (MMPs) family, which is involved in embryonic development, reproduction, tissue remodeling, as well as disease processes [24,25,26]. In the present study, we found that EGF could induce pituitary *MMP13* mRNA expression, but reduce *TIMP3* mRNA expression in grass carp pituitary cells. *TIMP3* is a member of tissue inhibitor of metalloproteinases (TIMP) gene family, which are the endogenous protein inhibitors of the MMPs family [27]. These results suggest that EGF could not only directly induce pituitary *MMP13* mRNA expression, but could further enhance *MMP13* expression via reducing its endogenous inhibitor *TIMP3* expression in grass carp pituitary. In addition, previous studies reported that the activated MMPs could release EGF from heparin-bound EGF (HB-EGF) [28]. Interestingly, our present study found that BB94, which was the inhibitor of MMPs, could partially suppress EGF-induced *EGR1* and *UTS1* mRNA expression in grass carp pituitary cells. Based on these results, it is reasonable for us to speculate that EGF-induced *MMP13* mRNA expression might be involved in the up-regulation of *UTS1* and *EGR1* mRNA expression by EGF in grass carp pituitary cells.

EGF receptors are transmembrane glycoprotein receptors containing an extracellular ligand-binding domain and an intracellular tyrosine kinase domain [29]. A previous study demonstrated that ErbB1 and ErbB2 have both been abundantly detected in normal pituitary corticotroph cells [30], but ErbB3 and ErbB4 were hardly detected in normal or tumoral corticotrophs [5]. Similarly, our previous study also found that both ErbB1 and ErbB2 were abundantly expressed in grass carp pituitary, but ErbB3 and ErbB4 were hardly detected in the pituitary [13]. In the present study, we found that EGF could induce *UTS1* and *EGR1* mRNA expression via the activation of both ErbB1 and ErbB2 in grass carp pituitary cells. Interestingly, EGF-regulated *MMP13* and *TIMP3* mRNA expression could only be mediated by ErbB1, but not ErbB2. Previous studies reported that ErbB1 could be activated by binding to growth factors of the EGF family [31]. However, ErbB2 has no ligand, it could bind with other activated ErbB receptors (ErbB1 or ErbB3) to form the highly active heterodimer [32,33]. Besides, the ErbB2 receptor could be activated by a ligand-independent mechanism, such as it could undergo the pH-dependent autophosphorylation [34]. These results suggest that EGF-induced pituitary *UTS1* and *EGR1* mRNA expression might be mediated by ErbB1/ErbB2 heterodimers, but EGF-regulated *MMP13* and *TIMP3* mRNA expression should be mediated by ErbB1 homodimers in teleost pituitary. For the post-receptor signaling pathway, our results found that EGF-induced *UTS1* and *EGR1* mRNA expression were coupled with the PI_3_K/AKT/mTOR and MEK_1/2_/ERK_1/2_ pathways. However, EGF-induced *MMP13* mRNA expression was only mediated by the MEK_1/2_/ERK_1/2_ pathway, but not PI_3_K/AKT/mTOR pathway. Similarly, a previous study also reported that only the MEK_1/2_/ERK_1/2_ pathway was involved in EGF-induced *MMP13* mRNA expression in gastric cancer cells [35]. These results, taken together, suggest that MEK_1/2_/ERK_1/2_ should be the critical signal transduction pathway in the up-regulation of *MMP13* by EGF. Recently, *MMP13* has emerged as a key target for the treatment of tumors [36]. These findings raise the possibility that MEK_1/2_ and ERK_1/2_ should be the critical signal transduction factors in EGF-induced tumors. In addition, it is confusing that the PI_3_K inhibitor Wortmannin could hugely induce *MMP13* mRNA expression in grass carp pituitary cells. We speculated that some factors in the pituitary could inhibit *MMP13* mRNA expression via the PI_3_K pathway, so the Wortmannin could induce pituitary *MMP13* expression through blocking these inhibitory actions.

**Figure 8:**
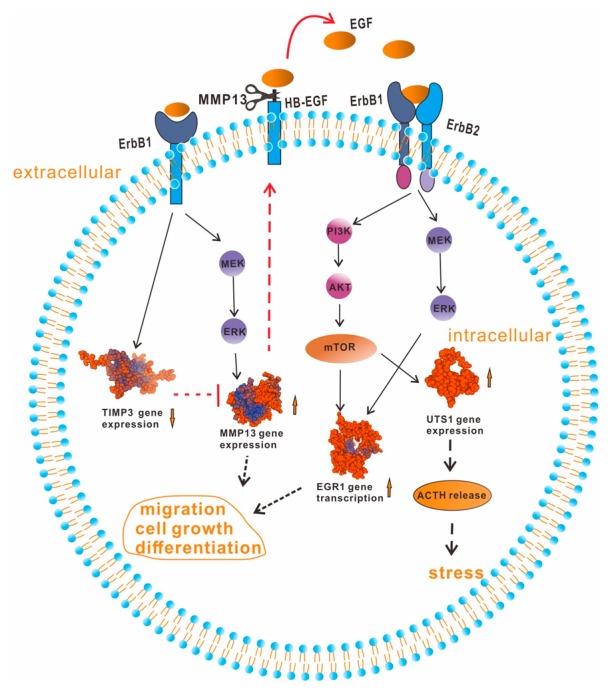
Working modal of EGF-induced *UTS1*, *EGR1*, *MMP13*, and *TIMP3* regulation in grass carp pituitary. EGF induced *UTS1* and *EGR1* mRNA expression were mediated by the PI_3_K/AKT/mTOR and MEK1/2/ERK1/2 pathways coupled with both ErbB1 and ErbB2. EGF-induced *MMP13* mRNA expression was only through the MEK1/2/ERK1/2 pathway coupled with ErbB1 and inhibited *TIMP3* mRNA expression via ErbB1. EGF-induced *MMP13* might be involved in the up-regulation of *UTS1* and *EGR1* mRNA expression by EGF in grass carp pituitary cells. The solid arrows indicated that the actions were verified by our study, the dotted arrows indicated that the actions were verified basing on the references. And the dotted “T” represented the inhibited action basing on the references.

In summary, our present study tried to examine the global pituitary actions of EGF in grass carp pituitary cells. Based on the transcriptomic analysis, EGF could significantly regulate 599 DEGs, which were involved in cell migration, cell differentiation, signal transduction, metabolic process, and phosphorylation. Then, we focused on three critical EGF-induced DEGs, namely *UTS1*, *EGR1*, and *MMP13*. Firstly, we found that EGF could significantly induce *UTS1* and *EGR1* mRNA expression via the activation of both ErbB1 and ErbB2 in grass carp pituitary cells. However, EGF-regulated *MMP13* and *TIMP3* mRNA expression were only mediated by ErbB1. The stimulatory actions of *UTS1* and *EGR1* mRNA expression were mediated by the PI_3_K/AKT/mTOR and MEK_1/2_/ERK_1/2_ pathways (Figure 8). The signaling mechanisms for *MMP13* responses were also similar, except that PI_3_K/AKT/mTOR was not involved. As we know, *MMP13* could release EGF from HB-EGF. In addition, our results found that the MMPs inhibitor BB94 could suppress EGF-induced *EGR1* and *UTS1* mRNA expression in grass carp pituitary cells. These results, taken together, suggest that EGF-induced *MMP13* mRNA expression might be involved in the up-regulation of *UTS1* and *EGR1* mRNA expression by EGF in grass carp pituitary cells (Figure 8).

## 4. Materials and Methods

### 4.1. Animals and Chemicals

One-year-old grass carps (1+) (*Ctenopharyngodon idellus*) with a body weight (BW) of 1.0–1.5 kg were bought from local markets and kept in the aquaria at 20 ± 2 °C for seven days and without feeding for at least three days prior to use in the experiment. To prepare the pituitary cells, grass carps were anesthetized in well-aerated water containing 0.05% MS-222 (Sigma, St. Louis, MO, USA) according to the protocol approved by the committee for animal use at Huazhong Agricultural University (Ethical Approval No. HBAC20091138; Date: 15 November 2009). Human EGF was purchased from GenScript Corporation (Nanjing, China) and dissolved in double-distilled deionized water and stored as 0.1 mM stocks in small aliquots at −80 °C. The pharmacological agents for receptor specificity and signal pathways (listed in Supplemental Table S1) were prepared as 10 mM frozen stocks in small aliquots and diluted with pre-warmed culture medium to appropriate concentrations 15 min prior to drug treatment.

### 4.2. Cell Culture, RNA Extraction and cDNA Library Construction

The grass carp pituitaries were rinsed three times with Hanks Balanced Salt Solution (HBSS; 400 mg KCl, 600 mg KH_2_PO4, 350 mg NaHCO_2_, 8 g NaCl, 48 mg Na_2_HPO_4_, and 1 g D-Glucose in 1 L ddH_2_O), and dispersed by trypsin/DNase II digestion method [37]. Then, grass carp pituitary cells were seeded in 24-well culture plates at a density of 2.5 × 10^6^ cells/well/mL at 28 °C under 5% CO_2_ for 15~18 h in plating medium. After that, the pituitary cells were incubated with EGF dissolved in testing medium for 24 h. Total RNA were harvested from the plate by adding 500 μL of Trizol reagent (Invitrogen, Carlsbad, CA, USA) to each well and shaking the plate for 10 min at 160~170 rpm on the shaker. The RNA was treated with DNase I to remove contaminating genomic DNA. The concentration and sample purity of total RNA were estimated using a Nanodrop 2000 spectrophotometer, and the quality of RNA was analyzed on an Agilent 2100 Bioanalyzer using the RNA 6000 Nano kit (Agilent Technologies, Santa Clara, CA, USA). Then, the RNA (RIN > 8.0) samples were sent to Majorbio Genome Center (Shanghai, China) for library preparation by TruSeq™ RNA sample prep Kit (Illumina, San Diego, CA, USA) and sequencing on HiSeq4000 (Illumina). A read depth of 600 million 150-bp single end reads was selected. An average of ~90% of the reads mapped to the grass carp genome (http://bioinfo.ihb.ac.cn/gcgd). All raw-sequence read data were deposited in NCBI Sequence Read Archive (SRA)2 with accession number SRP148383.

### 4.3. Differential Expression Genes (DEGs) Analysis and Functional Enrichment

Clean data could be obtained by removing read operations containing adapters, poly-N, and low-quality reads from the raw data. High-quality clean reads were mapped to the grass carp genome using TopHat v2.0 (http://ccb.jhu.edu/software/tophat/index.shtml). In different samples, gene expression levels were estimated by the number of fragments per kilobase transcript (FPKM). The read counts were further normalized into FPKM values. The fold changes were calculated by using RSEM software v 1.2.7 [38] and the DEGs were analyzed by using the R Bioconductor package, edgeR which calculated assuming a negative binomial distribution for the transcript level. The *p*-value was used to set the threshold for the differential gene expression test. The threshold of the *p*-value in multiple tests was determined by the value for the false discovery rate (FDR) [39]. DEGs were screened with a cut-off conditions of fold change (FC) > 1.5, *p* < 0.05 and FDR < 0.001. Functional annotation of gene ontology (GO) terms was analyzed by using Blast2GO software (https://www.blast2go.com/) [40], and GO functional classification of unigenes were analyzed by using WEGO 2.0 software (http://wego.genomics.org.cn/) [41]. Functional enrichment analysis, including GO and KEGG, was performed using Goatools (or KOBAS) software (https://github.com/tanghaibao/GOatools) [42].

### 4.4. Real-Time Quantitative PCR Validation

Grass carp pituitary cells were seeded in 24-well culture plates at a density of 2.5 million/mL/well and incubated with test substances for the duration as indicated. After drug treatment, the total RNA was isolated from pituitary cells by Trizol reagent (Invitrogen) and reversely transcribed by HifairTM III 1st Strand cDNA Synthesis Kit (gDNA digester plus) (Yeasen Biotech, Shanghai, China). After RNA isolated and reversely transcribed, the ABI 7500 real-time PCR system was used to detect the mRNA transcription of *MMP13*, *UTS1*, *EGR1*, and *TIMP3* with specific primers (see Supplementary Table S2 for primer sequences and PCR condition). In these experiments, plasmid DNA containing the gene coding sequence was subjected to gradient dilution as a standard for data calibration. In addition, parallel real-time PCR of β-actin was used as an internal control. The specific methods for dose- and time-dependent experiment, receptor specificity, signal transduction of EGF-induced *UTS1*, *EGR1* and *MMP13* mRNA expression could see Supplementary Methods in Supplemental Materials.

### 4.5. Data Transformation and Statistical Analysis

In this experiment, for real-time PCR of *MMP13*, *UTS1*, *EGR1*, and *TIMP3* mRNA, standard curves with dynamic range of 10^5^ and correlation coefficient > 0.95 were used for data calibration with ABI7500 software (Applied Biosystems, USA). *MMP13*, *TIMP3*, *EGR1*, and *UTS1* mRNA data were normalized with β-actin transcript level, and then were transformed as a percentage of the mean value in the control group without drug treatment (as “%Ctrl”). In the present study, the eight replicates (expressed as Mean ± SEM) were pooled results from two individual experiments prior to statistical analysis; all data were tested for normality of distribution using the Shapiro–Wilk normality test. One-way ANOVA and two-way ANOVA were used to test the significant difference according to different experiments. The differences between groups were considered as significant at *p* < 0.05 (“*”) or highly significant at *p* < 0.01 (“**”). The groups denoted by different letters represent a significant difference at *p* < 0.05.

## Figures and Tables

**Table 1:** Up-regulated genes by epidermal growth factor (EGF) in grass carp pituitary cells.

Gene	FC	*p*-Value	Description	Molecular Function
*DHX33*	1.81	1.39 × 10^−3^	DEAH-box helicase 33	ATP binding, helicase activity
*E2.7.3.2*	2.04	4.99 × 10^−8^	Creatine kinase M-type	ATP binding, kinase activity
*DCK*	1.74	8.70 × 10^−4^	Deoxycytidine kinase	ATP binding, nucleoside kinase activity
*MFGE8*	2.11	1.05 × 10^−38^	Rho GTPase-activating protein 10	Calcium ion binding
*KCNMA1*	1.7	3.82 × 10^−11^	Calcium-activated potassium channel subunit alpha	Calcium-activated potassium channel activity
*SGPP1*	1.85	5.09 × 10^−14^	Sphingosine-1-phosphate phosphatase 1	Catalytic activity
*COX6B*	1.9	1.39 × 10^−5^	Cytochrome c oxidase subunit 6B1	Cytochrome-c oxidase activity
*EGR1*	3.75	2.20 × 10^−151^	Early growth response protein 1	DNA binding,metal ion binding
*EDNRB*	1.74	1.01 × 10^−10^	Endothelin B receptor	Endothelin receptor activity
*ETV5*	1.7	7.60 × 10^−16^	ETS translocation variant 5	Equence-specific DNA binding
*FABP7*	2.93	9.81 × 10^−15^	Fatty acid-binding protein, brain	Fatty acid binding,transporter activity
*CDH1*	3.53	6.37 × 10^−7^	Cadherin-1	G-protein alpha-subunit binding
*RAB37*	2.12	5.92 × 10^−9^	Ras-related protein Rab-37	GTP binding
*RRAS2*	1.99	2.19 × 10^−9^	Ras-related protein R-Ras2	GTP binding
*ARHGAP10*	1.93	8.35 × 10^−11^	Rho GTPase-activating protein 10	Gtpase activator activity
*RGL2*	1.75	9.95 × 10^−28^	Ral guanine nucleotide dissociation stimulator	Guanyl-nucleotide exchange factor activity
*UTS1*	24.99	7.16 × 10^−149^	Urotensin1	Hormone activity
*Slα*	1.83	2.80 × 10^−49^	Somatolactin	Hormone activity
*PRL*	1.86	1.09 × 10^−51^	Prolactin	Hormone activity
*HasA*	1.79	7.90 × 10^−10^	Hyaluronan synthase 2	Hyaluronan synthase activity
*CDKAL1*	1.7	3.56 × 10^−2^	CDK5 regulatory subunit-associated protein 1-like 1	Kdo transferase activity
*PDE9*	2.32	9.56 × 10^−59^	High affinity cGMP-specific 3	Metal ion binding
*GALNT12*	1.79	2.06 × 10^−19^	Polypeptide N-acetylgalactosaminyltransferase 12	Metal ion binding, transferase activity
*MMP13*	289.6	0.00	Collagenase 3	Metalloendopeptidase activity
*NTRK3*	2.09	2.62 × 10^−6^	_	Neurotrophin receptor activity
*CADM4*	1.86	9.31 × 10^−32^	Cell adhesion molecule 4	Protein binding
*STK40*	1.97	2.60 × 10^−27^	threonine-protein kinase 40	Protein serine/threonine kinase activity
*Dusp14*	1.82	6.61 × 10^−18^	Dual specificity protein phosphatase 14	Protein tyrosine phosphatase activity
*Dusp2*	2.28	9.33 × 10^−18^	Dual specificity protein phosphatase 2	Protein tyrosine phosphatase activity
*Dusp4*	2.46	4.73 × 10^−16^	Dual specificity protein phosphatase 4	Protein tyrosine phosphatase activity
*Dusp5*	1.83	7.81 × 10^−6^	Dual specificity protein phosphatase 5	Protein tyrosine phosphatase activity
*Dusp7*	2.08	7.58 × 10^−31^	Dual specificity protein phosphatase 7	Protein tyrosine phosphatase activity
*OXSR1*	2.85	1.93 × 10^−14^	Serine-proteinkinase OSR1	Receptor signaling protein kinase activity
*CORIN*	5.22	4.15 × 10^−47^	Corin, serine peptidase	Serine-type endopeptidase activity
*SERPINB*	2.05	1.85 × 10^−3^	Leukocyte elastase inhibitor	Serine-type endopeptidase inhibitor activity
*KRT2*	2.14	5.43 × 10^−17^	Keratin, type II cytoskeletal 8	Structural molecule activity
*CPLX3_4*	1.7	1.02 × 10^−4^	Complexin-3	Syntaxin binding
*TPMT*	1.68	2.34 × 10^−3^	Thiopurine S-methyltransferase	Thiopurine S-methyltransferase activity
*FOSL1*	2.46	5.59 × 10^−47^	Fos-related antigen 1	Transcription factor activity
*BRA, T*	2	1.12 × 10^−6^	Brachyury protein homolog A	Transcription regulatory region DNA binding
*PTPRM*	1.78	3.30 × 10^−12^	Receptor-type tyrosine-protein phosphatase mu	Transmembrane receptor activity
*RNF144*	1.72	1.73 × 10^−10^	Probable E3 ubiquitin-protein ligase RNF144A-A	Tubulin-glycine ligase activity
*MYD88*	1.68	3.04 × 10^−13^	Myeloid differentiation primary response protein MyD88	Tyrosine kinase activity
*AVPR2*	1.94	6.54 × 10^−10^	Vasopressin V2 receptor	Vasopressin receptor activity
*GALT*	2.39	1.18 × 10^−3^	Galactose-1-phosphate uridylyltransferase	Zinc ion binding
*ZCCHC9*	1.74	1.44 × 10^−2^	Zinc finger CCHC domain-containing protein 9	Zinc ion binding, nucleic acid binding

FC: fold change.

**Table 2:** Down-regulated genes by EGF in grass carp pituitary cells.

Gene	FC	*p*-Value	Description	Molecular Function
*TER*	0.55	1.69 × 10^−5^	Very-long-chain enoyl-CoA reductase	Acting on the CH-CH group of donors
*ADCY6*	0.49	1.5 × 10^−20^	Adenylate cyclase type 6	Adenylate cyclase activity
*NRIP2*	0.50	8.4×10^−15^	Nuclear receptor-interacting protein 2	Aspartic-type endopeptidase activity
*SEK*	0.36	7.2 × 10^−35^	Ephrin type-A receptor 3	ATP binding
*Hsc70*	0.52	1.1 × 10^−21^	Heat shock cognate 70	ATP binding
*Hsp70*	0.55	6.03 × 10^−8^	Heat shock protein70	ATP binding
*CDH11*	0.38	2.2 × 10^−40^	Cadherin-11	Calcium ion binding
*CHP2*	0.51	0.000023	Calcineurin B homologous protein 1	Calcium ion binding
*PH-4*	0.50	6.3 × 10^−9^	Transmembrane prolyl 4-hydroxylase	Calcium ion binding
*E4.2.1.1*	0.53	2.3 × 10^−11^	Carbonic anhydrase 2	Carbonate dehydratase activity, zinc ion binding
*KCNC1*	0.51	4.1 × 10^−8^	_	Delayed rectifier potassium channel activity
*RYBP*	0.58	1.84 × 10^−13^	RING1 and YY1-binding protein A	DNA binding
*EIF2AK2*	0.57	1.5 × 10^−6^	Eukaryotic translation initiation factor 2-alpha kinase 2	Double-stranded RNA adenosine deaminase activity
*THBS1*	0.56	5.83 × 10^−23^	Thrombospondin-1	Extracellular matrix binding
*NTSR1*	0.50	7.1 × 10^−8^	Neurotensin receptor type 1	G-protein coupled neurotensin receptor activity
*RAB39B*	0.57	0.00158	Ras-related protein Rab-39B	GTP binding
*REM2*	0.44	6.6 × 10^−20^	GTP-binding protein REM 2	GTP binding
*RND3*	0.56	9.36 × 10^−7^	Rho-related GTP-binding protein RhoE	GTP binding
*RIG-I*	0.57	5.4 × 10^−8^	Probable ATP-dependent RNA helicase DDX58	Helicase activity, nucleic acid binding
*CYP4V*	0.29	6.2 × 10^−36^	Cytochrome P450 4V2	Heme binding, iron ion binding
*RAC3*	0.42	2 × 10^−7^	p21-Rac3; Flags: Precursor	Hydrolase activity
*LPL*	0.31	2.7×10^−55^	Lipoprotein lipase	Lipoprotein lipase activity
*GALNT13*	0.46	0.000016	Polypeptide GalNAc transferase 13	Metal ion binding
*DNAJA4*	0.54	1.1 × 10^−7^	DNA J homolog subfamily A member 4	Metal ion binding, heat shock protein binding
*GTF3A*	0.54	2.17 × 10^−10^	General transcription factor IIIA	Metal ion binding,nucleic acid binding
*PARP7S*	0.57	2.26 × 10^−6^	Poly (ADP-ribose) polymerase family, member 12b	Metal ion binding
*IFT54*	0.58	4.74 × 10^−7^	TRAF3-interacting protein 1	Microtubule binding
*TIMP3*	0.58	1.43 × 10^−13^	Tissue inhibitor of metalloproteinase 3	_
*ACTC1*	0.52	1.5 × 10^−10^	Actin, alpha cardiac muscle 1	Myosin binding
*DDX58*	0.54	0.00001	DEAD box protein 58	Nucleic acid binding
*NKTR*	0.53	4.2 × 10^−17^	NK-tumor recognition protein	Peptidyl-prolyl cis-trans isomerase activity
*MOX44*	0.53	2.8 × 10^−10^	CD53 molecule	Protein binding
*CCK4*	0.52	1.8 × 10^−13^	Protein tyrosine kinase 7	Protein tyrosine kinase activity
*SIAH1*	0.47	1.6 × 10^−11^	Siah E3 ubiquitin protein ligase 1	Protein-glycine ligase activity
*ITGB2*	0.41	1.9 × 10^−34^	Integrin beta-2	Receptor activity
*NOTCH*	0.53	7.4 × 10^−12^	Notch 1 extracellular truncation	Receptor activity, calcium ion binding
*NRP2*	0.36	3.3 × 10^−46^	Neuropilin-2	Semaphorin receptor activity
*NFKB1*	0.56	0.00635	Nuclear factor NF-kappa-B p105 subunit	DNA binding transcription factor activity
*IAT7E*	0.57	0.003	GalNAc alpha-2,6-sialyltransferase III	Sialyltransferase activity
*SLC1A3*	0.46	6 × 10^−43^	Excitatory amino acid transporter 1	Sodium:dicarboxylate symporter activity
*UGT*	0.47	4.9×10^−17^	UDP-glucuronosyltransferase 1-1	Transferase activity
*B4GALT3*	0.55	4.51 × 10^−7^	Beta-1,4-galactosyltransferase 3	Transferase activity, transferring glycosyl groups
*NTRK2*	0.52	7.5 × 10^−12^	NT-3 growth factors receptor	Transmembrane receptor protein tyrosine kinase activity
*SLC16A7*	0.36	7.2 × 10^−63^	Monocarboxylate transporter 2	Transmembrane transporter activity
*SV2*	0.43	6.1 × 10^−17^	Synaptic vesicle glycoprotein 2B	Transmembrane transporter activity
*RNF41*	0.33	3.2 × 10^−30^	Ligand of Numb protein X 4	Ubiquitin-protein transferase activity
*SFRP2*	0.43	9.4 × 10^−30^	Secreted frizzled-related protein 2	Wnt-protein binding
*AMZ2*	0.54	0.002	Archaemetzincin-2	Zinc ion binding

## Supplementary Materials

Supplementary materials can be found at https://www.mdpi.com/1422-0067/20/20/5172/s1.

## Abbreviations

ACTH	adrenocorticotropic hormone
Akt	protein kinase B
CRH	hypothalamic corticotropin releasing hormone
DEGs	differential expression genes
EGF	epidermal growth factor
EGFR	epidermal growth factor receptor
*EGR1*	early growth response 1
ERK	extracellular signal-regulated kinase
FSH	follicle-stimulating hormone
GH	growth hormone
GO	gene ontology
HB-EGF	Heparin-binding EGF-like growth factor
HPA	hypothalamus-pituitary-adrenal
IGF	insulin-like growth factor
KEGG	Kyoto Encyclopedia of Genes and Genomes
LH	luteinizing hormone
MEK	Methyl Ethyl Ketone
MAPK	mitogen-activated protein kinase
*MMP13*	matrix metallopeptidase 13
MMP9	matrix metallopeptidase 9
mTOR	mammalian target of rapamycin
PI_3_K	phosphatidylinositol-3-kinase
PRL	prolactin
SLα	somatolactin α
TACR3	tachykinin receptor 3
*UTS1*	urotensin 1
