# Supplementary material for: Novel Pituitary Actions of Epidermal Growth Factor: Receptor Specificity and Signal Transduction for UTS1, EGR1, and MMP13 Regulation by EGF
Source: Int J Mol Sci. 2019 Oct 18;20(20):5172. doi: 10.3390/ijms20205172 (PMC6829292; doi:10.3390/ijms20205172)
Supplement: Supplementary file 1 [file ijms-20-05172-s001.zip › Supplemental Materials.docx]

**Supplemental Materials**

Novel pituitary actions of epidermal growth factor: Receptor specificity and signal transduction for UTS1, EGR1 and MMP13 regulation by EGF

Qiongyao Hu, Shaohua Xu, Cheng Ye, Jingyi Jia, Lingling Zhou, Guangfu Hu*

College of Fisheries, Hubei Provincial Engineering Laboratory for Pond Aquaculture, Huazhong Agricultural University, Wuhan, China, 430070

***** Correspondence: huguangfu@mail.hzau.edu.cn; Tel.: +86-2787282113

**Supplemental Methods**

**Experiment 1#: Time course experiment of EGF (10nM) in grass carp pituitary cells (3h, 6h, 12h, 24h, 36h).**

Grass carp pituitary cells were prepared by trypsin/DNase digestion method. Pituitary cells obtained were seeded in 24-well cluster plate at a density of 2.5×10^6^ cells/ml/well and incubated for 15~18hr for recovery in plating medium with 5% FBS at 28℃ under 5% CO_2_ and saturated humidity. On the following day, culture medium was replaced with testing medium with the appropriate levels of test substances and the cells were allowed to incubate at 28℃ for the duration as indicated for individual experiment. After EGF(10nM)-treatment for 3h, 6h, 12h, 24h, 36h, total RNA was extracted from individual well using Trizol and reversely transcribed by HifairTM III 1^st^ Strand cDNA Synthesis Kit (gDNA digester plus) (Yeasen Biotech, Shanghai, China). Then, ABI 7500 real-time PCR system was used to detect the mRNA transcription of MMP13, UTS1, EGR1, and TIMP3 with specific primers, respectively. In these studies, serial dilutions of plasmid DNA containing the ORF of MMP13, UTS1, EGR1, and TIMP3 cDNA were used as the standards for data calibration. Parallel real-time PCR measurement of β-actin was also conducted in individual experiment to serve as the internal control.

**Experiment 2#: Receptor specificity of EGF (10nM)-induced MMP13, UTS1, and EGR1 mRNA expression in grass carp pituitary cells.**

Grass carp pituitary cells were firstly pretreated with AG1478 (1μM or 10μM) or AG879 (1μM or 10μM) for 30 min. Then, EGF (10 nM) were used to treat the pituitary cells together. After 24h treatment, total RNA was extracted from individual well using Trizol and reversely transcribed by HifairTM III 1^st^ Strand cDNA Synthesis Kit (gDNA digester plus) (Yeasen Biotech, Shanghai, China). Then, ABI 7500 real-time PCR system was used to detect the mRNA transcription of MMP13, UTS1, EGR1, and TIMP3 with specific primers, respectively.

**Experiment 3#: Signal transduction of EGF (10nM)-induced MMP13, UTS1, and EGR1 mRNA expression in grass carp pituitary cells.**

Grass carp pituitary cells were firstly pretreated with U0126 (1μM) or Wortmannin (1μM) for 30 min. Then, EGF (10 nM) were used to treat the pituitary cells together. After 24h treatment, total RNA was extracted from individual well using Trizol and reversely transcribed by HifairTM III 1^st^ Strand cDNA Synthesis Kit (gDNA digester plus) (Yeasen Biotech, Shanghai, China). Then, ABI 7500 real-time PCR system was used to detect the mRNA transcription of MMP13, UTS1, EGR1, and TIMP3 with specific primers, respectively.

**Experiment 4#: Western blot for signaling kinases**

Grass carp pituitary cells were seeded in poly-D-lysine coated 24-well culture plates at a density of 2.5×10^6^ cells/ml/well and incubated with drug treatment for the duration as indicated in individual experiments. For detection of phosphorylation ERK and total ERK, the duration of drug treatment was reduced to 30 min based on time course validation. After drug treatment, culture medium from individual well was removed, and remaining cells were rinsed with PBS and lysed in RIPA buffer (50 mM Tris.HCl, 150 mM NaCl, 1 mM EDTA, 1% NP-40, and 0.25% Na deoxycholate) containing a final concentration of 1 × protease/phosphatase inhibitor cocktail (Roche). The cells lysate was cleared by high-speed centrifugation at 4℃, and the clear supernatant were resolved in 10% gel by SDS-PAGE. The antibodies that can be detected the phosphorylated and total p-ERK (1:5,000), and t-ERK (1:1,000), respectively, were used at the dilutions recommended by the manufactures. Following the incubation, the membranes were washed three times to remove non-specific binding of primary antibodies and the HRP-conjugated secondary antibodies [goat anti-rabbit IgG (1:5,000)] were introduced for signal development. Chemiluminescence signals for target immune-reactivity were detected using SuperSignal West Pico (PIERCE, Rockford) as the substrate and quantified using the IC440 CF Digital Science Image Station (Eastman Kodak). In these experiments, Western blot of β-actin was used as an internal control using its antibody (1:15,000; Oncogen, Cambrige, MA).

**Results and Discussion**

**Experiment 1#: Time course experiment of EGF (10nM) in grass carp pituitary cells (3h, 6h, 12h, 24h, 36h).**

In this experiment, the results showed that EGF (10nM) could significantly induce pituitary UTS1 (Supplemental Figure S1A) and MMP13 (Supplemental Figure S1C) mRNA expression in the time-dependent manner. In addition, EGF could inhibit the pituitary TIMP3 mRNA expression in the time-course dependent manner (Supplemental Figure S1D). Interestingly, EGF (10nM) could significantly induce EGR1 mRNA expression from 3h to 24h, but the rise in EGF-induced EGR1 mRNA expression was deceased after 36h in grass carp pituitary cells (Supplemental Figure S1B). Similarly, previous study found that EGF could induce the EGR-1 promoter activity in ECV304 cells (1). A transient rise in EGR1 promoter activity was noted during the 4-14 h of EGF treatment, but the upregulation of EGR1 promoter was decreased after 26 h (1).

**Supplemental Figure S1. Time course experiment of EGF (10nM) in grass carp pituitary cells (3h, 6h, 12h, 24h, 36h).** In the time course experiment, (A) EGF induced UTS1 mRNA expression in grass carp pituitary cells. (B) EGF induced EGR1 mRNA expression in grass carp pituitary cells. (C) EGF induced MMP13 mRNA expression in grass carp pituitary cells. (D) EGF inhibited TIMP3 mRNA expression in grass carp pituitary cells. The differences between groups were considered as significant at P<0.05 (“*”) or highly significant at P<0.01 (“**”).

**Experiment 2#: Receptor specificity of EGF (10nM)-induced MMP13, UTS1, and EGR1 mRNA expression in grass carp pituitary cells.**

In the receptor specificity experiment, EGF (10nM)-induced UTS1 and EGR1 mRNA expression could be abolished by both ErbB1 antagonist AG1478 (1μM or 10μM) (Supplemental Figure S2A,B) and ErbB2 antagonist AG879 (1μM or 10μM) (Supplemental Figure S2E,F), respectively. In addition, the EGF (10nM)-induced MMP13 mRNA expression could also be blocked by ErbB1 antagonist AG1478 (1μM or 10μM) (Supplemental Figure S2C), but could not ErbB2 antagonist AG879 (1μM or 10μM) (Supplemental Figure S2G) and without any changes of β-actin mRNA expression (Supplemental Figure S2D,H). These results suggested that EGF could induce UTS1 and EGR1 mRNA expression via activation of both ErbB1 and ErbB2 in grass carp pituitary cells. However, EGF-induced MMP13 mRNA expression could only be mediated by ErbB1, but not ErbB2.

**Supplemental Figure S2. Receptor specificity of EGF (10 nM)-induced UTS1, EGR1 and MMP13 mRNA expression.** Effects of ErbB1 antagonist AG1478 (1μM) or AG1478 (10 μM) on EGF (10nM)-induced UTS1 (A), EGR1 (B) MMP13 (C), and β-actin (D) mRNA expression for 24h, respectively. Effects of ErbB2 antagonist AG879 (1μM) or AG879 (10 μM) on EGF (10nM)-induced UTS1 (E), EGR1 (F) MMP13 (G), and β-actin (H) mRNA expression for 24h, respectively. The groups denoted by different letters represent a significant difference at P<0.05.

**Experiment 3#: Signal transduction of EGF (10nM)-induced** **MMP13, UTS1, and EGR1 mRNA expression in grass carp pituitary cells.**

To clarify the signal transduction for EGF-induced UTS1, EGR1 and MMP13, mRNA expression in grass carp pituitary cells, a pharmacological approach was used. The results showed that the upregulation of UTS1 and EGR1 mRNA expression by EGF (10nM) could be blocked by lower dose MEK inhibitor U0126 (1μM) (Supplemental Figure S3A,C), or lower dose PI3K inhibitor Wortmannin (1μM) (Supplemental Figure S3B,D), respectively. The stimulatory effects of EGF on MMP13 mRNA expression were significantly inhibited by simultaneous incubation with the MEK1/2 inhibitor U0126 (1 μM) (Supplemental Figure S3E), but not PI3K inhibitor Wortmannin (1 μM) (Supplemental Figure S3F). These results suggested that lower dose EGF (10nM)-induced UTS1 and EGR1 mRNA expression were coupled with PI3K and MEK1/2 pathways. However, EGF-induced MMP13 mRNA expression was only mediated by MEK1/2 pathway, but not PI3K pathway.

**Supplemental Figure S3. Signal transduction of EGF (10nM) -induced UTS1, EGR1 and MMP13 mRNA expression in grass carp pituitary cells.** Effects of 24-hr cotreatment with the MEK inhibitor U0126 (1 μM) or PI3K inhibitor Wortmannin (1 μM) on EGF (10nM)-induced UTS1 (A, B), EGR1 (C, D) and MMP13 (E, F) mRNA expression. After drug treatment, total RNA was isolated for real-time PCR of UTS1, EGR1 and MMP13 mRNA expression. The groups denoted by different letters represent a significant difference at P<0.05.

**Experiment 4#: Western blot for signaling kinases**

To confirm whether MEK/ERK cascades were involved in EGF-induced post-receptor signaling, the effects of EGF and EGFR inhibitor AG1478 treatment on ERK phosphorylation were tested in grass carp pituitary cells. As shown in Supplemental Figure S4, EGF could significantly induce the phosphorylation of ERK in grass carp pituitary cells. In addition, EGFR inhibitor AG1478 could significantly block EGF-induced ERK phosphorylation. These results suggested that the MEK/ERK cascades were involved in EGF-induced post-receptor signaling.

**Supplemental Figure S4. EGF-induced protein phosphorylation of ERK.** The grass carp pituitary cells were treated with 500 nM EGF or 10μM EGFR inhibitor AG1478 for 30 min. After that, cell lysate was prepared for Western blot by using the antisera for phosphorylated ERK and total ERK (A). Parallel blotting of β-actin was used as an internal control. The quantified graph is the representative blots in each group (B). Data presented are expressed as mean ± SEM (n = 3). The groups denoted by different letters represent a significant difference at P<0.05.

**Reference**

1. Tsai, J. C.; Liu, L.; Guan, J.; Aird, W. C. The Egr-1 gene is induced by epidermal growth factor in ECV304 cells and primary endothelial cells. *Am J Physiol Cell Physiol*, **2000**, 279(5), C1414-1424.
