# Supplementary material for: Novel Pituitary Actions of Epidermal Growth Factor: Receptor Specificity and Signal Transduction for UTS1, EGR1, and MMP13 Regulation by EGF
Source: Int J Mol Sci. 2019 Oct 18;20(20):5172. doi: 10.3390/ijms20205172 (PMC6829292; doi:10.3390/ijms20205172)
Supplement: Supplementary file 1 [file ijms-20-05172-s001.zip › Supplemental Tables .doc]

**Supplemental Table S1.** Primers used for quantitative real-time PCR

| **qPCR primers** | **Forward primer** | **Reverse primer** | **Length** | **Annealin**  **Tm** |
| --- | --- | --- | --- | --- |
| MMP13 | TGAAGAGACGCTGGATGTGATG | GCTGTGCCGCTGTAGATTCG | 230 | 60℃ |
| EGR1 | CGGCAGAAGGACAAGAAG | GGTGGTGACAGGAGAACT | 303 | 60℃ |
| TIMP3 | CTGAAGGAGCAGGTGGTG | GCAGATACTGGTCGTGATT | 271 | 56℃ |
| UTS1 | GACCACCCACATCCCCCTGAGC | CGGTTCAGTTCCGCCTGTTCCC | 365 | 56℃ |
| β-Actin | CTGGTATCGTGATGGACTCT | AGCTCATAGCTCTTCTCCAG | 280 | 56℃ |

**Supplemental Table S2.** The information for the drugs used in receptor specificity and signal transduction.

| **Item** | **Function** | **Manufacture** | **Cat. No.** |
| --- | --- | --- | --- |
| AG1478 | ErbB1 inhibitor | MCE | HY-13524 |
| AG879 | ErbB2 inhibitor | MCE | HY-20878 |
| AG1024 | IGF1-R inhibitor | MCE | HY-10253 |
| U0126 | MEK1/2 inhibitor | MCE | HY-12031 |
| LY3214996 | ERK1/2 inhibitor | MCE | HY-101494 |
| SB 203580 | p38MAPK inhibitor | MCE | HY-10256 |
| Wortmannin | PI3K inhibitor | MCE | HY-10197 |
| MK 2206 | Akt inhibitor | MCE | HY-10358 |
| Rapamycin | mTOR inhibitor | MCE | S1039 |
